# Pre-revascularization coronary wedge pressure as marker of adverse long-term left ventricular remodelling in patients with acute ST-segment elevation myocardial infarction

**DOI:** 10.1038/s41598-018-20276-6

**Published:** 2018-01-30

**Authors:** Mãdãlin Constantin Marc, Adrian Corneliu Iancu, Camelia Diana Ober, Cãlin Homorodean, Şerban Bãlãnescu, Adela Viviana Sitar, Sorana Bolboacã, Ioana Mihaela Dregoesc

**Affiliations:** 1 “Iuliu Haţieganu’’ University of Medicine and Pharmacy, 8 Victor Babeş, Cluj-Napoca, Romania; 2 “Niculae Stãncioiu” Heart Institute, Department of Cardiology, 19-21 Calea Moţilor, Cluj-Napoca, Romania; 3 “Carol Davila” University of Medicine and Pharmacy, 37 Dionisie Lupu, Bucharest, Romania

## Abstract

The aim of this study was to investigate the relationship between coronary wedge pressure (CWP), measured as a marker of pre-procedural microvascular obstruction, and left ventricular remodelling in high-risk ST-segment elevation myocardial infarction (STEMI) patients. Pre-revascularization CWP was measured in 25 patients with high-risk anterior STEMI. Left ventricular volumes and ejection fraction were echocardiographically measured at discharge and at follow-up. A 20% increase in left ventricular volumes was used to define remodelling. Patients with CWP ≤ 38 mmHg were characterized by late ventricular remodelling. Patients with CWP > 38 mmHg developed a progressive remodelling process which was associated with a significant 60 months increase in left ventricular volumes (P = 0.01 for end-systolic volume and 0.03 for end-diastolic volume) and a significant decrease in left ventricular ejection fraction (P = 0.05). A significant increase in both left ventricular end-systolic (P = 0.009) and end-diastolic volume (P = 0.02) from baseline to 60 months follow-up was recorded in patients with extracted thrombus length ≥2 mm. Pre-revascularization elevated CWP was associated with increased left ventricular volumes and decreased ejection fraction at long-term follow-up. CWP was a predictor of severe left ventricular enlargement, besides extracted thrombus quantity.

## Introduction

Infarct size, microvascular obstruction (MVO) and most probably inflammation are important determinants of left ventricular remodelling after acute ST-segment elevation myocardial infarction (STEMI). Left ventricular remodelling is an important factor in the development of heart failure and a predictor of mortality^1–3^.

Detection and treatment of MVO during acute STEMI is of the utmost importance since it frequently occurs even after timely culprit artery revascularization^1–3^.

None of the prophylactic and therapeutic approaches available are effective for MVO treatment^3,4^. Recently, it has also been observed that intracoronary pressure measurement is significantly influenced by the presence and severity of MVO in STEMI. Moreover, it can predict the final extent of global and regional irreversible myocardial injury and left ventricular function at long term follow-up^5^.

On the other hand, patients with high collateralization, defined as visible collaterals on the coronary angiogram or as Rentrop scores 1–3, had a 36% reduced all-cause mortality risk compared with patients with Rentrop score 0^6^. Pressure measurements can also define collateral flow. There are some controversies regarding collateral flow and pressure measurements in coronary arteries affected by MVO in acute myocardial infarction^7,8^.

The mean pressure distal to the occlusion is nothing other than the coronary wedge pressure (CWP) and depends on collateral flow, which is usually modest, especially if no Rentrop collaterals are seen. High CWP measured after reperfusion was proven to be related to MVO in STEMI patients^7–9^.

The aim of this study was to determine whether elevated CWP, measured as a marker of pre-procedural MVO, correlated with left ventricular remodelling in high-risk STEMI patients.

## Results

**Figure 1:**
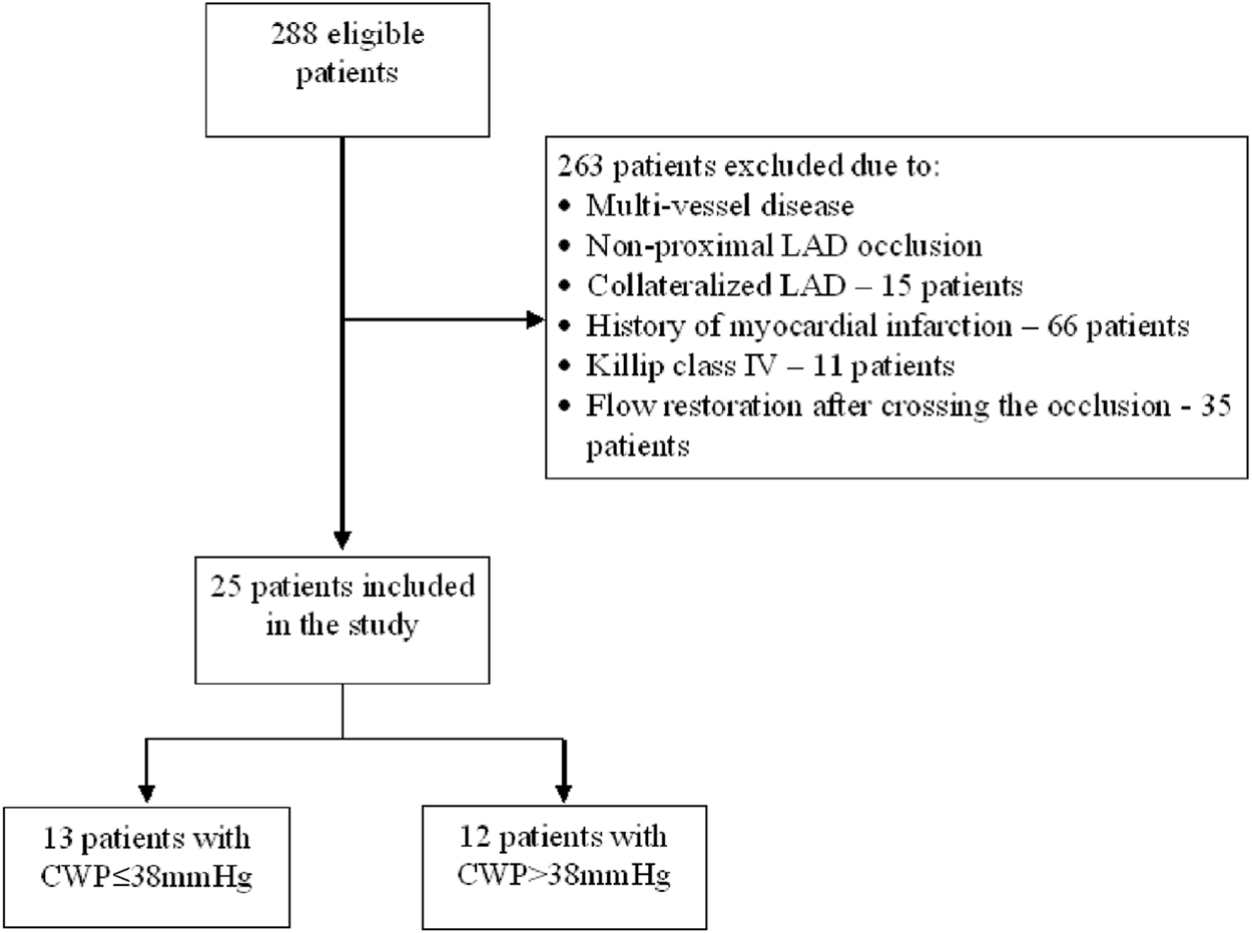
Flowchart. CWP = coronary wedge pressure; LAD = left anterior descending artery.

25 patients were included in the final analysis (Fig. 1). All patients had a proximally non-collateralized occluded LAD, which was successfully opened in all cases.

**Figure 2:**
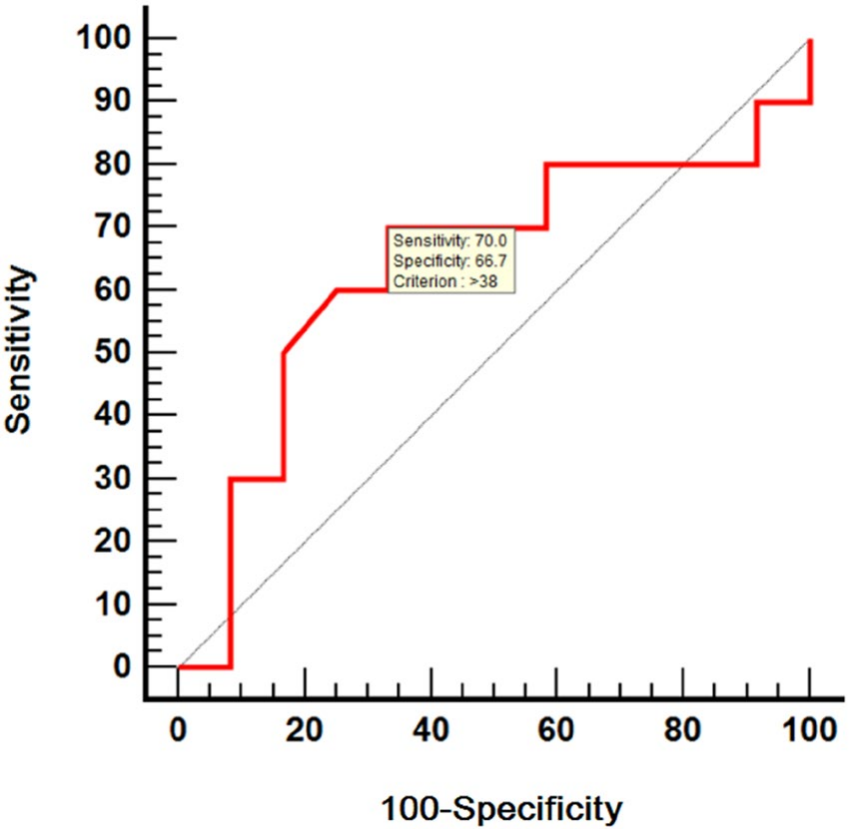
Receiver operating characteristic curves for coronary wedge pressure in predicting left ventricular end-systolic volume ≥75 ml at 60 months follow-up. AUC = area under the curve.

ROC curve analysis was used to assess the performance of CWP in identifying patients with overtime LVESV increase. The AUC was 0.637 (P = 0.25) for detecting ≥75 ml LVESV at 60 months follow-up, with an optimal cut-off >38 mmHg. This value returned a sensitivity of 70% and a specificity of 66.7% (Fig. 2). The 75 ml value selected for LVESV is 20% above the upper limit of normal, as defined by transthoracic echocardiography.

The patients were divided into two groups according to CWP value. Group A consisted of 13 patients with CWP ≤ 38 mmHg, while Group B consisted of 12 patients with CWP > 38 mmHg.

### Baseline characteristics

The baseline demographic, clinical and non-clinical characteristics are presented in Tables 1 and 2.

**Table 1:** Clinical and laboratory characteristics of the two groups.

	GROUP A, n = 13 (CWP ≤ 38 mmHg)	GROUP B, n = 12 (CWP > 38 mmHg)	P-value
**Presentation**			
Age (yrs.), m ± SD	56.69 ± 11.60	53.91 ± 14.98	0.60
Sex (male), no (%)	10 (77)	10 (83.33)	0.92
TIT (min), m ± SD	230.38 ± 74.73	412.92 ± 225.17	0.02
Door to balloon (min), m ± SD	62.30 ± 20.87	71.25 ± 58.50	0.62
**Risk Factors**			
BMI (kg/m^2^), m ± SD	28.98 ± 5.07	29.09 ± 2.38	0.94
Diabetes (yes), no (%)	5 (38.46)	4 (33.33)	0.88
Hypertension (yes), no (%)	8 (61.53)	7 (58.33)	0.76
Smokers (yes), no (%)	8 (61.53)	5(41.66)	0.72
**Laboratory Parameters**			
Leukocytes (/mm^3^), m ± SD	12870.83 ± 3421.64	11389 ± 2136.20	0.24
Glycaemia (mg/dl), m ± SD	148.16 ± 36.11	137.2 ± 46.40	0.50
Creatinine clearance (ml/min), m ± SD	105.51 ± 30.26	120.99 ± 42.95	0.30
CK-MB (U/l), median (Q1-Q3)	260.00 (145–441)	198.00 (83.75–360.25)	0.47

BMI = body mass index; CK-MB = creatine-kinase myocardial band; CWP = coronary wedge pressure; m = mean; Q1 = first quartile; Q3 = third quartile; SD = standard deviation; TIT = total ischemic time.

**Table 2:** Non-clinical characteristics of the two groups.

PCI Characteristics			
**Extracted thrombus, %**			
**<**0.5 mm	38.46	8.33	
0.5–2 mm	61.53	33.32	
**>**2 mm	0	58.33	0.001
Stent length (mm), m ± SD	20.30 ± 4.09	21.91 ± 4.14	0.33
Stent diameter (mm), m ± SD	3.35 ± 0.30	3.19 ± 0.27	0.20
**Echocardiographic Baseline Characteristics**			
LVEF (%), m ± SD	48.30 ± 9.36	54.5 ± 9.47	0.11
LVESV (ml), m ± SD	57 ± 27.93	44.16 ± 11.95	0.20
LVEDV (ml), m ± SD	106.38 ± 40.56	99.5 ± 33.94	0.87

CWP = coronary wedge pressure; LVEF = left ventricular ejection fraction; LVESV = left ventricular end-systolic value; LVEDV = left ventricular end-diastolic value; m = mean; PCI = percutaneous coronary intervention; SD = standard deviation.

Except for TIT, which proved significantly higher in Group B as compared to Group A (p = 0.02), the two groups were similar with regard to presentation characteristics and cardiovascular risk factors. Baseline laboratory and angiographic investigated parameters were not significantly different between groups, with the exception of aspirated thrombotic material. The extracted thrombus length was in most of the cases between 0.5 and 2 mm for Group A and >2 mm for group B, the percentage of cases with thrombus length >2 mm being significantly higher in Group B (P = 0.001).

Each patient received at least one stent, with no significant difference between groups with regard to stent length or diameter.

The two groups were comparable with regard to baseline left ventricular echocardiographic parameters.

### Clinical follow-up

During the 60 months follow-up, there were three cases of target vessel revascularization, for in-stent restenosis, one in group A and two in group B. All of them were treated with drug eluting stents and none of the interventions were associated with peri-procedural myocardial infarction. There was one case of acute STEMI caused by a right coronary artery occlusion in a patient in group A, while another patient from the same group presented with aggravated exertional angina and required revascularization for a “de novo” right coronary artery stenosis. Regarding overall repeat revascularization, the difference recorded between groups did not reach significance (P = 0.74).

### Follow-up left ventricular echocardiographic parameters

**Figure 3:**
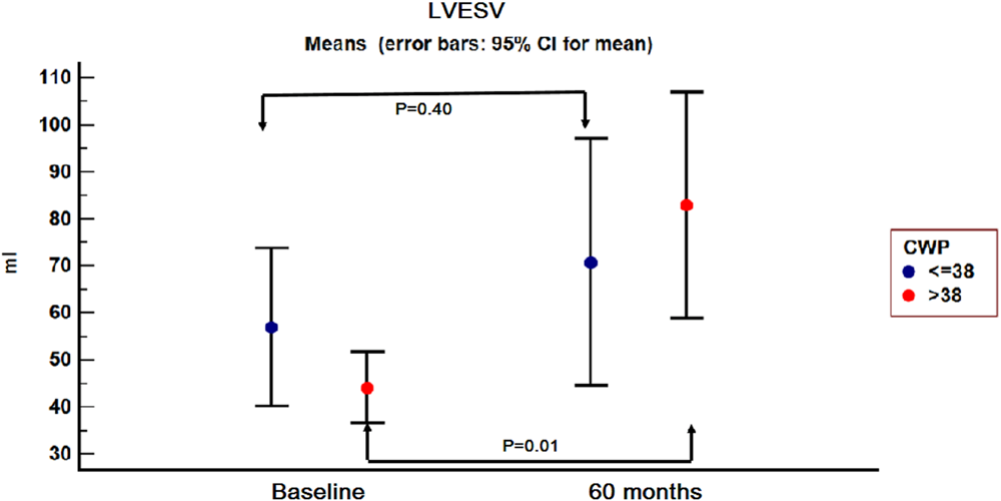
Left ventricular end-systolic volume evolution in the two groups from baseline to 60 months follow-up. 95% CI = 95% confidence interval; CWP = coronary wedge pressure; LVESV = left ventricular end-systolic volume.

**Figure 4:**
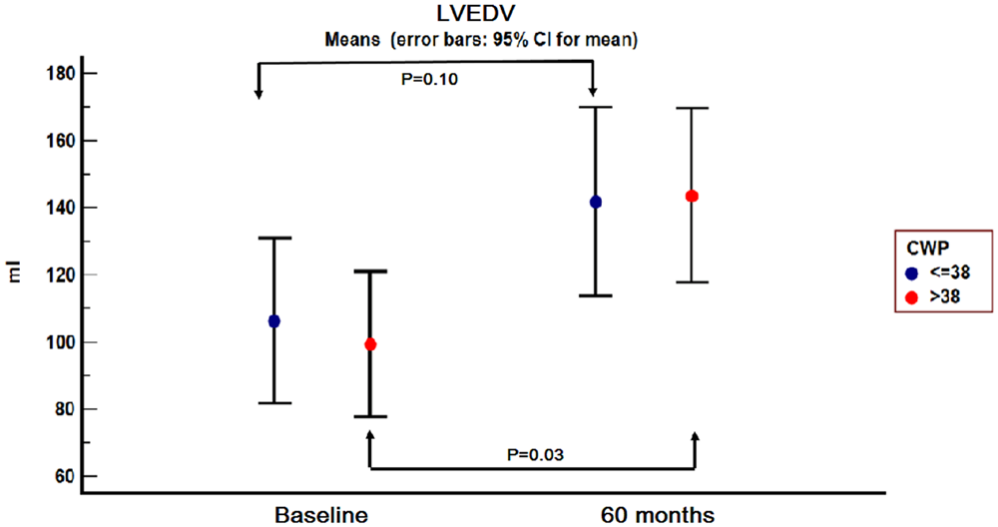
Left ventricular end-diastolic volume evolution in the two groups from baseline to 60 months follow-up. 95% CI = 95% confidence interval; CWP = coronary wedge pressure; LVEDV = left ventricular end-diastolic volume.

**Figure 5:**
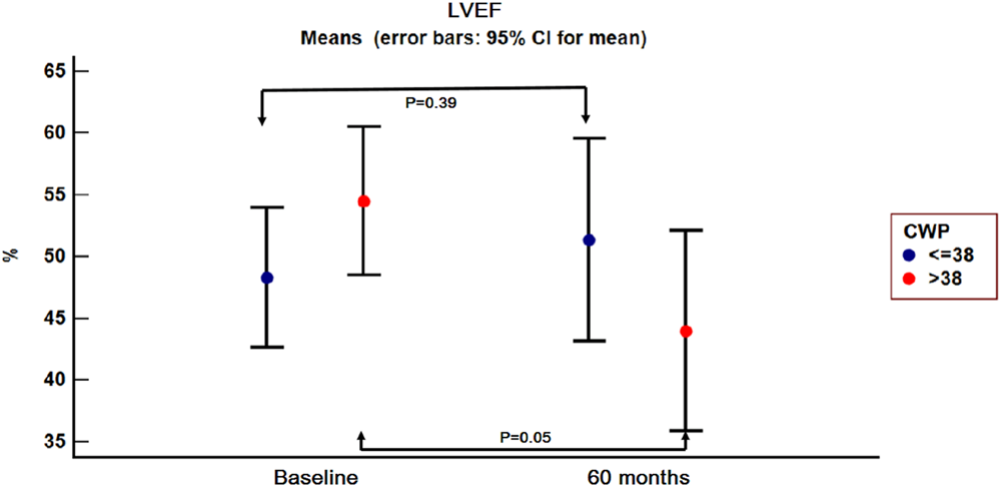
Left ventricular ejection fraction evolution in the two groups from baseline to 60 months follow-up. 95% CI = 95% confidence interval; CWP = coronary wedge pressure; LVEF = left ventricular ejection fraction.

The patients were reassessed at one, six, 24 and 60 months respectively. At the 24 and 60 months follow-up, three and respectively two patients from group A did not present, while one patient from group B died.For group B, there was a significant increase in LVESV starting at the 24 months follow-up visit (P = 0.02), while the increase in LVEDV and the decrease in LVEF were not significant at that moment. However, at 60 months follow-up, there was a significant increase in LVESV (P = 0.01) and LVEDV (P = 0.03) compared to baseline and a 10% decrease in mean LVEF (P = 0.05) (Figs 3, 4 and 5).

Regarding group A, the 60 months increase in LVESV and LVEDV was not significant (P = 0.40 and 0.10 respectively), nor was the 5% increase in mean LVEF (P = 0.39).

64% of patients in group A had a LVEF greater than 50% at the 60 months follow-up as compared to 27% of patients in group B (P = 0.07).

**Figure 6:**
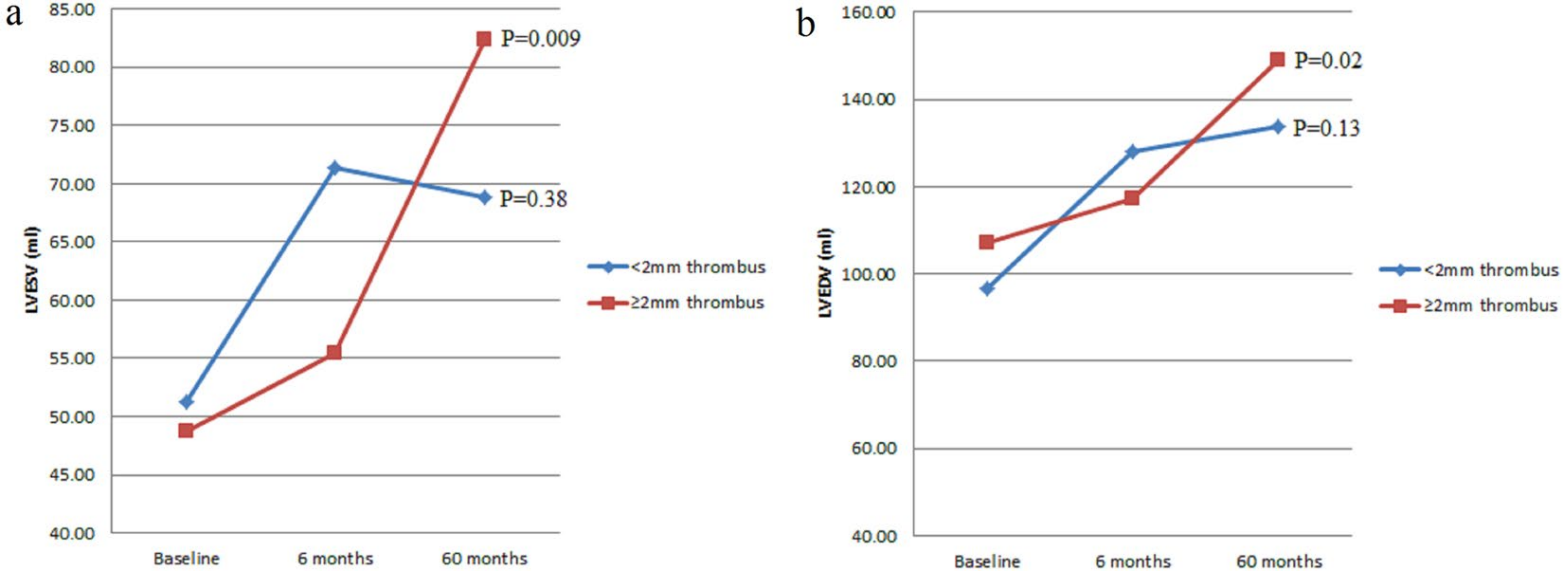
Left ventricular end-systolic (Panel a) and end-diastolic volume (Panel b) evolution from baseline to 60 months follow-up, according to extracted thrombus length. LVESV = left ventricular end-systolic volume; LVEDV = left ventricular end-diastolic volume.

A significant increase in both LVESV (P = 0.009) and LVEDV (P = 0.02) from baseline to 60 months follow-up was recorded in patients with extracted thrombus length ≥2 mm. The 60 months increase in LVESV and LVEDV, while still present, did not reach statistical significance in patients with extracted thrombus length <2 mm (P = 0.38 and 0.13 respectively) (Fig. 6, Panel a and b).

### Left ventricular remodelling

**Figure 7:**
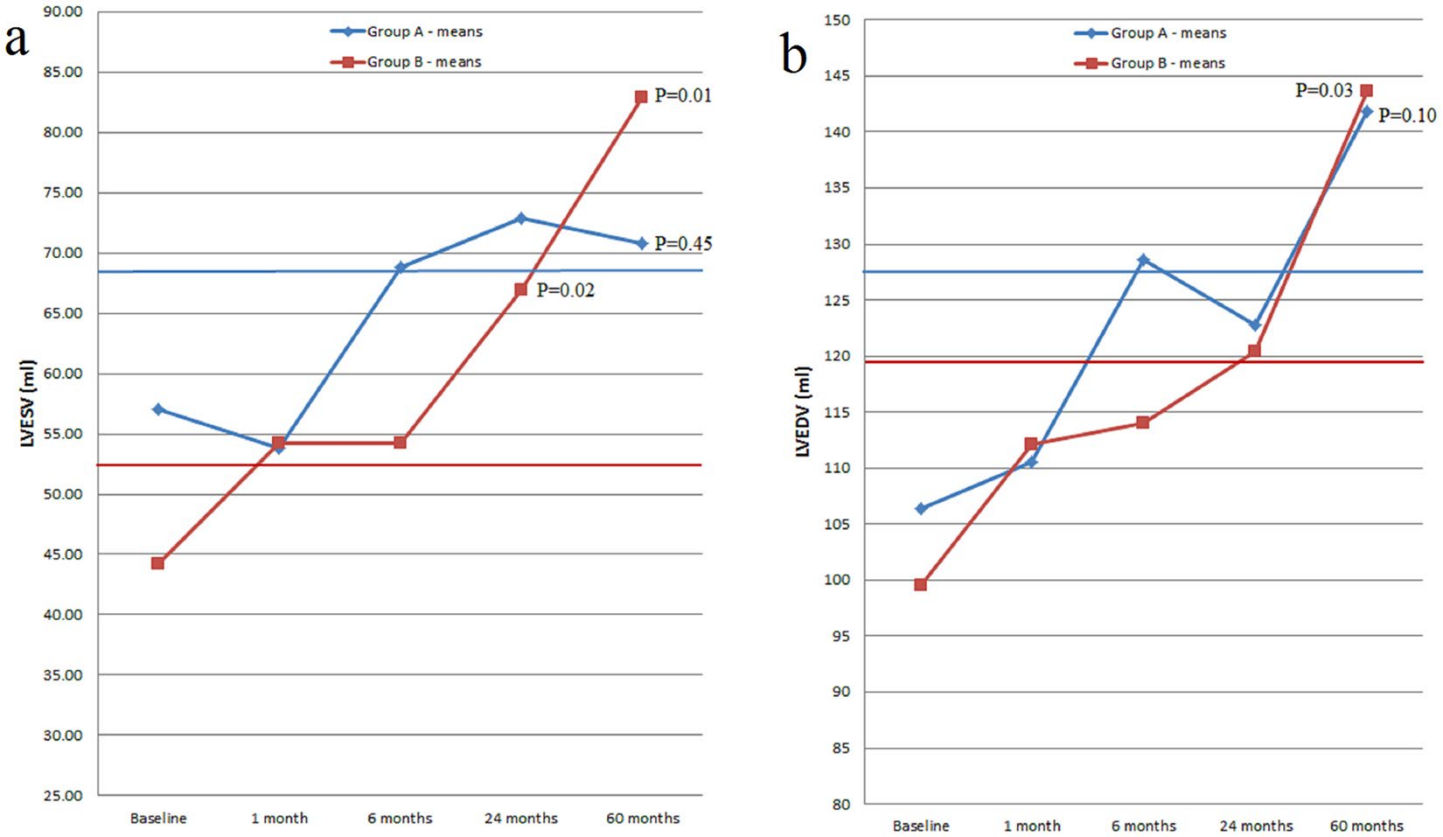
Left ventricular end-systolic volume (Panel a) and left ventricular end-diastolic volume (Panel b) progression in the two groups, from baseline to 60 months follow-up, with left ventricular remodelling criteria. Horizontal blue line −20% increase limit in Group A; horizontal red line −20% increase limit in Group B; LVESV = left ventricular end-systolic; LVEDV = left ventricular end-diastolic volume.

A “progressive dilation” pattern was recorded in Group B. In this group, a 22.82% increase in LVESV was observed at the one month follow-up visit. The increase reached 51.48% at 24 months, while at 60 months the increase in the same parameter reached 87.70%. Regarding LVEDV, the 60 months increase reached 44.36% (Fig. 7, Panel a and b).

In Group A, “a late dilation” pattern was observed. The 20% LVESV increase limit was reached after 6 months, while at 60 months follow-up the increase reached 24.24% (Fig. 7, Panel a). In this group LVEDV increased with 33.31% at 60 months follow-up (Fig. 7, Panel b).

In the study population TIT was independent of left ventricular remodelling, both at 6 months (342.5 ± 223.00 vs. 295.38 ± 150.49, P = 0.54) and at 60 months follow-up (332 ± 211.28 vs. 313.12 ± 183.98 min, P = 0.82). The same finding was recorded in each of the two groups taken separately. Moreover, TIT did not vary according to CWP in patients with 6 months left ventricular remodelling (236.66 ± 82.62 min in Group A vs. 448.33 ± 275.13 min in Group B, P = 0.12). However, TIT was significantly longer in patients with CWP > 38 mmHg and left ventricular remodelling at 60 months follow-up as compared to those with CWP ≤ 38 mmHg and left ventricular remodelling at 60 months follow-up (426.87 ± 238.25 vs. 206.66 ± 57.85 min, P = 0.03). In this context, at 60 months follow-up, LVESV increased with only 24.24% in Group A as compared to 87.70% in group B. The 60 months LVESV increase recorded in Group B was 3.61 times greater than the one recorded in Group A. Data available in Supplementary Table S1.

## Discussion

In our study, on long term follow-up, adverse left ventricular remodelling was observed in both groups of patients, irrespective of CWP, but its pattern and especially its magnitude differed.

Group A, with CWP ≤ 38 mmHg, was characterized by “late ventricular remodelling”, but the increase in left ventricular volumes from baseline to 60 months follow-up was not significant. However, in this group, the 5% improvement in LVEF recorded at 60 months follow-up was considered significant and reverse remodelling was considered to be present, as described by Rizzello^10^.

Patients in Group B, defined by CWP > 38 mmHg, suffered a “progressive remodelling”. This process was associated with a significant increase in left ventricular volumes and a significant decrease in left ventricular ejection fraction, as shown in Figs 3–5 and Fig. 7. The strict inclusion criteria (proximal LAD occlusion, non-collateralized vessel and absent flow after guidewire and Twin-Pass catheter insertion) led from the very beginning to the selection of patients with a high risk for death and heart failure^6,11^. Infarct size was comparable in both groups, irrespective of CWP value, while TIT significantly differed between the two groups.

Short time to reperfusion has been shown to be a powerful predictor of a good outcome in STEMI patients. However, the recorded 182 min difference in TIT between groups, although statistically significant, could not explain the different pattern and magnitude of left ventricular remodelling. On long term follow-up LVESV increase recorded in Group B was 3.61 times greater than the one recorded in Group A. Moreover, in group B left ventricular remodelling started at one month and evolved progressively. In group A, the 20% threshold was reached only after 6 months, and it did not record a considerable increase during the next 4.5 years. TIT was significantly longer in patients with 60 months left ventricular remodelling only when associated with CWP > 38 mmHg.

In a study by Kramer *et al*.^12^, patients with extracted older thrombus had significantly longer TIT as compared to those with fresh thrombus. No differences were observed for postprocedural TIMI flow and enzymatic infarct size. At 4 years follow-up, all-cause mortality was 2-fold higher in patients with older thrombus as compared to patients with fresh thrombus. Kramer demonstrated that TIT was a predictor for survival only when associated with thrombus age. Similarly, in our study TIT was predictor of long term unfavourable outcome only when associated with high coronary wedge pressure. An analysis of pooled data from randomized trials^13^ showed a linear association of mortality with longer TIT in patients with thrombolytic therapy but not with PCI. This finding questions the traditionally accepted relationship between the opened coronary artery, myocardial salvage, and left ventricular remodelling. In this context, the old concept of “illusion of reperfusion”^14^ may gain new meaning.

Regarding aspirated thrombi in patients with distal embolization and MVO, these are larger and older as compared to those aspirated in patients without MVO^15,16^. The embolism that occurs before symptoms’ onset in patients with STEMI was recently suggested by Kramer^12^ and described by De Maria *et al*.^17^. In this context, high CWP describes pre-procedural MVO, an important determinant of left ventricular remodelling. In our study, a large quantity of extracted thrombus and higher CWP correlated with the theory developed by Kramer^12^. Kramer hypothesized that patients with older thrombus may have experienced short temporary occlusive thromboses before admission. Repeated episodes of spontaneous recanalization could therefore be associated with more thrombus burden, extensive embolism and MVO. In this scenario, the inflammatory shower resulting from inflammatory cell-rich old thrombus^18^ determines pre-procedural MVO. Pre-procedural MVO, characterized by an important inflammatory component, precedes intra-procedural microvascular embolization.

The patients with Rentrop collateralization were excluded because of the controversies regarding the increase in pressure by means of collateral flow.

There are no data in literature regarding a cut-off for CWP in predicting poor outcome after STEMI revascularization. Data regarding pre-procedural CWP measurements are also missing. An interesting issue concerns the cut-off for pressure at zero flow (Pzf). Patel *et al*.^5^ demonstrated that Pzf is the most sensitive invasive coronary physiology index currently available for assessing microcirculation after primary PCI and for predicting myocardial injury and left ventricular function at 6 months. In their study, Pzf was significantly better at predicting MVO and myocardial injury than the index of microcirculatory resistance. CWP might be similar in some circumstances to Pzf both of them being pressure measurements at ceased coronary flow. The 38 mmHg cut-off derived for CWP, very close to the 42 mmHg cut-off derived for Pzf, is further proof of their similarity in predicting left ventricular remodelling.

This was a small, single-centre study, but it was the first study to directly examine and compare the effects of pre-procedural CWP on left ventricular remodelling in patients with acute myocardial infarction. Cardiac magnetic resonance imaging, the gold standard in the assessment of MVO, interstitial oedema, left ventricular volumes, ejection fraction and infarct size, was not available in our study group. Both Pzf and CWP are connected to interstitial oedema.

Another limitation is represented by the small sample size, a common issue with most of the studies addressing these issues. The studied sample consists of less than 10% of the potentially eligible patients. However, this was mainly due to the strict inclusion criteria. The decision to include only patients with proximal LAD occlusion and single vessel-disease was taken in order to ensure the homogeneity of the studied population. Moreover, patients with previous myocardial infarction were excluded and so were patients with collateralized LAD, in order to avoid any influence of collateral flow on the CWP value. TIMI flow was checked after the occlusion was crossed with the dual-lumen catheter, and only patients with TIMI flow zero were included in the study. The consistency of the relationship between CWP and multiple important left ventricular echocardiographic parameters, with highly significant P-values, suggests that these findings are of clinical relevance and could therefore be safely extrapolated to other vascular territories.

TIMI flow restoration after vessel wiring or the presence of a circulated culprit vessel could limit this technique. However, the use of over-the-wire balloons and pressure measurement after balloon inflation are viable solutions to these issues. Damping of distal coronary pressure could result as a consequence of the small inner lumen of the catheter. On the other hand, the microembolization produced as the catheter crosses the occluded segment may influence CWP especially by increasing its values.

Further investigation is justified in order to confirm the hereby generated hypotheses and to define the clinical utility of CWP in patients with acute STEMI. Moreover, larger studies are needed in order to fully characterize the relationship between pre-procedural CWP and the classic parameters of MVO. Since TIT has lost its intrinsic prognostic value in STEMI patients, and thrombus aspiration tends to be abandoned, CWP may become an important parameter in defining the extent of future left ventricular remodelling.

## Methods

This was a prospective, observational, single-centre study. Between November 1, 2010 and December 31, 2011, 288 consecutive patients referred to “Niculae Stãncioiu” Heart Institute in Cluj-Napoca for a first episode of anterior STEMI were screened for inclusion. The inclusion criteria were typical ischemic ongoing pain within 12 h of onset, ST segment elevation of at least 0.2 mV in two contiguous electrocardiographic leads and acutely totally occluded and non-collateralized proximal left anterior descending artery (LAD) with Thrombolysis in Myocardial Infarction (TIMI) 0 flow. The exclusion criteria were previous myocardial infarction, multi-vessel disease, Killip class IV, LAD collateralization and restoration of TIMI flow at any time before pressure measurement.

The investigators performing the echocardiographic examinations and the statistical analysis were blind with regard to the group the patients belonged to. The study complies with the 1975 Declaration of Helsinki. The research protocol was approved by “Niculae Stãncioiu” Heart Institute Ethics Committee and all patients gave written informed consent.

All patients received standard double anti-platelet and anticoagulant therapy according to the practice guidelines. 325 mg of aspirin and 600 mg of clopidogrel were administered at the first medical contact, while heparin was given during the interventional procedure at a dose of 70 U/kg.

After crossing the occlusion with the wire, a double lumen 5200 Model Twin-Pass catheter (Vascular Solutions, Minneapolis, Minn., USA) was passed through the lesion. Its position was checked with a small diluted dye injection. CWP was measured through the catheter if TIMI flow remained zero. CWP was recorded after saline flushing of the catheter. CWP measurement, followed by downstream eptifibatide administration, was fulfilled in a maximum of two minutes. Eptifibatide infusion was performed in accordance with a previously mentioned protocol in order to achieve a high distal drug concentration^19^. The aim was to achieve the maximum therapeutic effect of a glycoprotein IIbIIIa inhibitor on microcirculation. Primary PCI was then performed using manual thrombus aspiration (Export Aspiration Catheter; Medtronic, Inc., Minneapolis, Minn., USA) followed by direct stent implantation in all cases.

Total ischemic time (TIT), defined as the time from the onset of symptoms to Twin-Pass catheter insertion was recorded in all patients.

Aspirated material was classified according to combined fragments length as follows: small (<0.5 mm), moderate (0.5–2 mm), or large (>2 mm)^4^.

Blood samples were obtained on admission and at 6, 12, 18, 24, 48 and 72 h after the procedure and creatine-kinase myocardial band (CK-MB) peak-value was determined in order to estimate the extent of necrosis.

Left ventricular end-systolic volume (LVESV), end-diastolic volume (LVEDV), and the ejection fraction (LVEF) were measured using the Simpson method at discharge, and at one, 6, 24 and 60 months follow-up. The mean value of five measurements was calculated for each echocardiographic examination. A 20% increase in left ventricular volumes was used to define remodelling according to the classification used by Rizzello and Bolognese^10,20^.

At discharge patients received double antiplatelet therapy with aspirin and clopidogrel for one year. Treatment with aspirin, beta-blockers, statins, and angiotensin-converting-enzyme inhibitors was administered indefinitely. Blood pressure was monitored at the follow-up visits. No significant differences were recorded between the two groups. Patients with symptomatic heart failure and LVEF below 35% were treated with aldosterone receptor antagonists according to the practice guidelines. None of the patients received an implantable cardioverter defibrillator.

The corresponding author had full access to all the data in the study and takes responsibility for its integrity and the data analysis.

### Statistical methods

Data distribution was assessed using Kolmogorov – Smirnov and D’Agostino tests. Quantitative continuous data were summarized as mean ± standard deviation whenever data proved normally distributed; otherwise, median and interquartile range (Q1‒Q3), where Q1 = first quartile and Q3 = third quartile, were used. The groups were compared with Student t-test or Wilcoxon rank-sum test, as appropriate. Categorical data were presented as percentages.

ANOVA, Kruskal-Wallis and Friedman tests were used to analyse repeated measures of LVEF and left ventricular volumes. Receiver-operating characteristic (ROC) curves were constructed to evaluate the accuracy of CWP in predicting the LVESV changes. The area under the ROC curves (AUC) was determined as a scalar measure of performance. The Youden index was used to identify the ideal cut-off values from the ROC curves. Statistical analysis was performed with MedCalc (v 10.3.0.0, MedCalc Software, Ostend, Belgium). A two-sided P-value < 0.05 was considered statistically significant.

### Data availability statement

supporting data available as Supplementary Dataset.

## Conclusion

Our study measured pre-procedural CWP as a parameter of pre-procedural MVO and evaluated its relationship with left ventricular remodelling in a group of high-risk anterior acute STEMI patients. Pre-revascularization elevated CWP was associated with increased left ventricular volumes and decreased ejection fraction at long-term follow-up. CWP was a predictor of severe left ventricular enlargement, besides extracted thrombus quantity.

## Impact on Daily Practice

The current prophylactic and therapeutic measures for left ventricular remodelling in the setting of acute STEMI are either controversial or inadequate. Through CWP measurement a prognostic issue is achieved and meantime an important therapeutic method is created: “the downstream intracoronary drug delivery”.

In the infarcted wall, MVO could impede coronary blood ejection into the venous circulation. In this scenario, CWP is increased by means of a large and tall systolic wave^21^. It suggests embolism and MVO and identifies the group of patients most likely to benefit from intracoronary glycoprotein IIb/IIIa inhibitors or thrombolysis.

On the other hand, CWP is also influenced by interstitial pressure as interstitial oedema compresses capillaries and increases the intravascular pressure^22^. A new coronary wave flow configuration occurs, with continuous elevation of the pressure line. Probably these patients would benefit more from anti-inflammatory therapy^23^.

Besides, the use of micro-catheters before reperfusion can define the thrombotic burden and the length of the lesion by small dye injection. This simple procedure allows for direct stenting by avoiding pre-dilatation in an era when thrombus aspiration failed to prove any benefit.

## Electronic supplementary material

Supplementary Table S1

## References

[1] Hamirani YS, Wong A, Kramer CM, Salerno M (2014). Effect of microvascular obstruction and intramyocardial haemorrhage by CMR on LV remodelling and outcomes after myocardial infarction: a systematic review and meta-analysis. J. Am. Coll. Cardiol. Img..

[2] Weir RA (2010). Microvascular obstruction remains a portent of adverse remodelling in optimally treated patients with left ventricular systolic dysfunction after acute myocardial infarction. Circ. Cardiovasc. Imaging..

[3] Eitel I (2014). Intracoronary Compared With Intravenous Bolus Abciximab Application During Primary Percutaneous Coronary Intervention in ST-Segment Elevation Myocardial Infarction: Cardiac Magnetic Resonance Substudy of the AIDA STEMI Trial. J. Am. Coll. Cardiol..

[4] Svilaas T (2008). Thrombus aspiration during primary percutaneous coronary intervention. N. Engl. J. Med..

[5] Patel N (2015). Zero-Flow Pressure Measured Immediately After Primary Percutaneous Coronary Intervention for ST-Segment Elevation Myocardial Infarction Provides the Best Invasive Index for Predicting the Extent of Myocardial Infarction at 6 Months: An OxAMI Study (Oxford Acute Myocardial Infarction). J. Am. Coll. Cardiol. Intv..

[6] Meier P (2012). The impact of the coronary collateral circulation on mortality: a meta-analysis. Eur. Heart. J..

[7] Yamamoto K (2001). Pressure-derived collateral flow index as a parameter of microvascular dysfunction in acute myocardial infarction. J. Am. Coll. Cardiol..

[8] Sezer M (2006). Pressure-Derived Collateral Flow Index: A Strong Predictor of Late Left Ventricular Remodeling After Thrombolysis for Acute Myocardial Infarction. Coron. Artery. Dis..

[9] Sezer M (2004). New support for clarifying the relationship between ST-segment resolution and microvascular function: degree of ST-segment resolution correlates with the pressure-derived collateral flow index. Heart..

[10] Rizzello V (2004). Opposite patterns of left ventricular remodelling after coronary revascularization in patients with ischemic cardiomyopathy: role of myocardial viability. Circulation..

[11] Valgimigli M (2011). Persistent coronary no flow after wire insertion is an early and readily available mortality risk factor despite successful mechanical intervention in acute myocardial infarction: a pooled analysis from the STRATEGY (Single High-Dose Bolus Tirofiban and Sirolimus-Eluting Stent Versus Abciximab and Bare-Metal Stent in Acute Myocardial Infarction) and MULTI- STRATEGY (Multicenter Evaluation of Single High-Dose Bolus Tirofiban Versus Abciximab With Sirolimus-Eluting Stent or Bare- Metal Stent in Acute Myocardial Infarction Study) trials. J. Am. Coll. Cardiol. Intv..

[12] Kramer MC (2008). Presence of older thrombus is an independent predictor of long-term mortality in patients with ST-elevation myocardial infarction treated with thrombus aspiration during primary percutaneous coronary intervention. Circulation..

[13] Zijlstra F (2002). Clinical characteristics and outcome of patients with early (<2 h), intermediate (2–4 h) and late (>4 h) presentation treated by primary coronary angioplasty or thrombolytic therapy for acute myocardial infarction. Eur. Heart J..

[14] Lincoff AM, Topol EJ (1993). Illusion of reperfusion: does anyone achieve optimal reperfusion during acute myocardial infarction?. Circulation..

[15] Yunoki K (2013). Relationship of Thrombus Characteristics to the Incidence of Angiographically Visible Distal Embolization in Patients With ST-Segment Elevation Myocardial Infarction Treated With Thrombus Aspiration. J. Am. Coll. Cardiol. Intv..

[16] Fokkema ML (2009). Incidence and clinical consequences of distal embolization on the coronary angiogram after percutaneous coronary intervention for ST-elevation myocardial infarction. Eur. Heart J..

[17] De Maria GL (2015). How does coronary stent implantation impact on the status of the microcirculation during primary percutaneous coronary intervention in patients with ST-elevation myocardial infarction?. Eur. Heart J..

[18] Yunoki K (2012). Erythrocyte-rich thrombus aspirated from patients with ST-elevation myocardial infarction: association with oxidative stress and its impact on myocardial reperfusion. Eur. Heart J..

[19] Iancu A, Ober C, Bondor CI, Cadiş H (2012). Microvascular effect of intracoronary eptifibatide in acute myocardial infarction. Cardiology..

[20] Bolognese L (2002). Left Ventricular Remodeling After Primary Coronary Angioplasty. Circulation..

[21] Sezer M (2007). Relationship between microvascular resistance and perfusion in patients with reperfused acute myocardial infarction. J. Interv. Cardiol..

[22] Echavarría-Pinto M (2015). Use of intracoronary physiology indices in acute coronary syndromes. Interventional Cardiology..

[23] Westman PC (2016). Inflammation as a Driver of Adverse Left Ventricular Remodeling After Acute Myocardial Infarction. J. Am. Coll. Cardiol..

