# Supplementary material for: Pre-revascularization coronary wedge pressure as marker of adverse long-term left ventricular remodelling in patients with acute ST-segment elevation myocardial infarction
Source: Sci Rep. 2018 Jan 30;8:1897. doi: 10.1038/s41598-018-20276-6 (PMC5789971; doi:10.1038/s41598-018-20276-6)
Supplement: Supplementary file 1 — Supplementary Table S1 [file 41598_2018_20276_MOESM1_ESM.doc]

**Full title:** Pre-revascularization coronary wedge pressure as marker of adverse long-term left ventricular remodelling in patients with acute ST-segment elevation myocardial infarction

**Authors:** 1. Mãdãlin Constantin Marc^1, 2^, MD, 2. Adrian Corneliu Iancu ^* 1, 2^, MD, PhD, 3. Camelia Diana Ober^2^, MD, 4. Cãlin Homorodean^1^, MD, PhD, 5. Şerban Bãlãnescu^3^, MD, Ph.D, 6. Adela Viviana Sitar^1^, MD, 7. Sorana Bolboacã^1^, MD, PhD, 8. Ioana Mihaela Dregoesc^1, 2^, MD.

**Affiliations:** 1. “Iuliu Haţieganu” University of Medicine and Pharmacy, 8 Victor Babeş, Cluj-Napoca, Romania; 2. “Niculae Stãncioiu” Heart Institute, Department of Cardiology, 19-21 Calea Moţilor, Cluj-Napoca, Romania; 3. “Carol Davila” University of Medicine and Pharmacy, 37 Dionisie Lupu, Bucharest, Romania

*** Corresponding author:**

Prof.Adrian C. Iancu, MD, PhD

19-21 Calea Moţilor, Cluj-Napoca, 400001, Romania

Telephone number: +40744751027; Fax number: +40264595090

E-mail address: [adrian_iancu@hotmail.com](mailto:adrian_iancu@hotmail.com)

| **Supplementary Table S1** | | | |  | | | | | |
| --- | --- | --- | --- | --- | --- | --- | --- | --- | --- |
| Baseline and 60 months follow-up left ventricular ejection fraction and left ventricular volumes in the studied group. | | | | | | | |  | |
| LVEF=left ventricular ejection fraction; LVEDV=left ventricular end-diastolic volume; LVESV=left ventricular end-systolic volume. | | | | | | |  | |  |
| CWP | Baseline LVEF | 60 months LVEF | Baseline LVEDV | 60 months LVEDV | Baseline LVESV | 60 months LVESV |
| 18 | 56 | 32 | 133 | 208 | 81 | 146 |
| 23 | 35 | 60 | 201 | 133 | 130 | 53 |
| 25 | 53 | 30 | 81 | 177 | 49 | 115 |
| 26 | 40 | 55 | 135 | 60 | 58 | 26 |
| 27 | 54 | 60 | 75 | 143 | 34 | 53 |
| 27 | 53 | 60 | 65 | 80 | 30 | 22 |
| 29 | 52 |  | 106 |  | 51 |  |
| 30 | 45 | 55 | 145 | 148 | 80 | 73 |
| 30 | 66 |  | 62 |  | 21 |  |
| 35 | 50 | 40 | 123 | 175 | 60 | 116 |
| 36 | 31 | 68 | 71 | 149 | 49 | 50 |
| 38 | 50 | 55 | 110 | 150 | 55 | 68 |
| 38 | 43 | 50 | 76 | 137 | 43 | 57 |  | | |
| 42 | 35 | 40 | 177 | 122 | 25 | 79 |  | |  |
| 45 | 50 |  | 85 |  | 44 |  |  |
| 46 | 47 | 57 | 111 | 125 | 67 | 54 |  |
| 52 | 56 | 58 | 70 | 140 | 31 | 59 |  |
| 52 | 56 | 32 | 110 | 179 | 49 | 114 |  |
| 55 | 62 | 35 | 129 | 195 | 38 | 121 |  |
| 70 | 45 | 50 | 77 | 165 | 40 | 91 |  |
| 76 | 59 | 56 | 127 | 66 | 51 | 30 |  |
| 78 | 62 | 50 | 81 | 162 | 38 | 94 |  |
| 90 | 63 | 21 | 79 | 188 | 30 | 149 |  |
| 92 | 50 | 35 | 50 | 128 | 30 | 78 |  |
| 104 | 69 | 50 | 98 | 110 | 60 | 43 |  |
